# A Manual of Histology

**Published:** 1883-07

**Authors:** 


**A Manual of Histology.** Edited and prepared by THOMAS E. SATTERTHWAITE, M. D., Professor of Histological and Pathological Anatomy in the New York Post Graduate Medical College; Pathologist to the St. Luke's and Presbyterian Hospitals. Second edition, enlarged and revised, containing 202 illustrations, with an appendix. New York: Wm. Wood & Co., No. 56 and 58 Lafayette Place.

When the first edition of this work appeared it was received with almost unanimous favor by the reviewers, and the profession have added their marked commendation by so soon exhausting the first edition. The changes in this volume are necessarily few, for the first edition was well up with the knowledge of the day, and but little has been added in the short time that has elapsed since its publication. Some textual errors have been corrected, and an appendix added, giving the latest contributions on the subjects of "The Lymphatic System" and "The Salivary Glands." We anticipate that a third edition of the book will be soon called for.

